# PEA-CLARITY: 3D molecular imaging of whole plant organs

**DOI:** 10.1038/srep13492

**Published:** 2015-09-02

**Authors:** William M. Palmer, Antony P. Martin, Jamie R. Flynn, Stephanie L. Reed, Rosemary G. White, Robert T. Furbank, Christopher P. L. Grof

**Affiliations:** 1 School of Environmental and Life Sciences, University of Newcastle, Callaghan, NSW, 2308, Australia; 2 School of Biomedical Sciences and Pharmacy, University of Newcastle, Callaghan, NSW 2308, Australia; 3 CSIRO Agriculture, Black Mountain, ACT, 2601, Australia; 4 ARC Centre of Excellence for Translational Photosynthesis, Australian National University, Acton, ACT, 2601, Australia

## Abstract

Here we report the adaptation of the CLARITY technique to plant tissues with addition of enzymatic degradation to improve optical clearing and facilitate antibody probe penetration. Plant-Enzyme-Assisted (PEA)-CLARITY, has allowed deep optical visualisation of stains, expressed fluorescent proteins and IgG-antibodies in Tobacco and Arabidopsis leaves. Enzyme treatment enabled penetration of antibodies into whole tissues without the need for any sectioning of the material, thus facilitating protein localisation of intact tissue in 3D whilst retaining cellular structure.

Fixation and embedding of plant tissue for molecular interrogation using techniques such as histological staining, immunohistochemistry or *in situ* hybridisation has been the foundation of cell biology studies for decades. Applying these techniques for 3D tissue analysis is seriously limited by the need to section the tissue, image each section, and then reassemble the images into a 3D representation of the structures of interest. Here we present a fundamental shift from the two dimensional plane to that of three dimensions whilst retaining molecular structures of interest without the need to section the plant tissue. Recent advances in fixation and ‘clearing’ techniques such as SeeDB^1^, Sca*l*eA2^2^, 3DISCO^3^, CLARITY^4^ and its recent variant PACT^5^ enabled intact imaging of whole embryos, brains and other organs in mouse and rat models. The new CLARITY system fixes and binds tissues within an acrylamide mesh structure. Proteins and nucleic acids are covalently linked to the acrylamide mesh by formaldehyde, then optically interfering lipid structures of animal cell membranes are removed using detergent (SDS). This renders such tissue optically transparent and suitable for deep imaging of up to ~5 mm using confocal microscopy^4^.

Three dimensional imaging of plants using confocal microscopy has been limited to already semi-transparent tissue types such as root tips or meristems but resolution becomes limiting in cells deeper within tissues^6^. Other plant specific imaging techniques including modified pseudo-Schiff propidium iodide (mPS-PI) staining do allow for deep optical penetration, although the clearing steps also remove proteins and nucleic acids^7^,^8^. A major hindrance to applying techniques such as CLARITY to plant material is the cell wall, comprised mainly of cellulose, hemicellulose, lignin and pectin, which is permeable only to molecules under 60 kDa^9^. This creates a significant permeability barrier as common IgG antibodies used in immunohistochemistry are ~150 kDa in size and therefore unable to penetrate cell walls. PEA-CLARITY overcomes this limitation by using cell wall degrading enzymes to increase wall permeability, together with starch hydrolysing enzymes to reduce optical interference from starch grains. Cell wall degrading enzymes have been used to achieve 3D immunofluorescence within *A. thaliana* apical meristems however with harsh enzymatic degradation, the tissue lost structural integrity^10^. A contrasting method using urea as clearing agent, together with enzyme treatment, generated 3D structural images of plant tissues to localise nuclei and cell walls simultaneously. They also retained fluorescence from transiently expressed mTalin-citrine, revealing intact actin microfilaments deep within cleared tobacco leaves^11^. Nevertheless, this protocol used sectioned pea root nodules to aid antibody penetration and could only use short enzyme treatments to avoid structural damage to the tissue. The PEA-CLARITY technique described here combines hydrogel fixation, tissue clearing and enzymatic degradation, allowing visualisation of expressed fluorescent proteins, including GFP and CFP, simultaneously with immunofluorescence labelling using common IgG antibodies in whole, intact tissues, without loss of structural integrity.

PEA-CLARITY was applied to leaf tissue from two model species, *A. thaliana* and *N. tabacum* (Fig. 1A), initially to confirm that molecular structures remained intact during fixation, hydrogel polymerization and clearing. During the fixation and clearing process the leaf tissue became transparent due to the removal of lipids, chlorophyll and other pigments (Fig. 1B). Following clearing, cell walls and starch were enzymatically degraded to allow passage of antibodies during immunolabelling and to slightly increase transparency of the tissue (Fig. 1C).

Tobacco leaf tissue fixed in hydrogel and cleared in SDS but not enzymatically degraded was stained with propidium iodide (PI, red-nucleus) and calcofluor white (CW, green—cell wall) to assess the retention of DNA and cellular structure (Fig. 2). Using CLSM the whole, intact tobacco leaf disc was imaged and the z-stacks were assembled into a raw, unprocessed 3D slice reconstruction (Fig. 2B). Optical penetration through the entire leaf thickness (~150 μm) was achieved. Vascular, epidermal, palisade mesophyll and spongy mesophyll cell structure was maintained with all nuclei fixed within the cytosol of the cell. Even long trichome protrusions remained perpendicular to the epidermal surface layer across the entire leaf disc (see supplementary 1 for basal trichome orientation; full trichomes were not imaged). Furthermore the staining of nuclei by PI demonstrated the retention of nuclear DNA. This technique also allowed deep optical penetration into the vascular bundle with nucleus of the sieve element / companion cell (SE/CC) clearly defined without the need for sectioning or epidermal peeling.

Mature leaves from *A. thaliana* lines stably transformed with GFP localised to the endoplasmic reticulum (C16251; ER-GFP) or with CFP localised to peroxisomes (C16259; Px-CFP)^12^, were CLARITY treated to assess the retention of endogenous fluorescent proteins. Strong ER-GFP and Px-CFP was observed throughout cleared *A. thaliana* leaves, including the internal leaf phloem at a depth of ~80 μm (Fig. 3).

Mature leaves from the *N. tabacum* stably transformed line, Sv-40, with GFP localised to nuclei^13^ was processed using the PEA-CLARITY treatment, incubated with cell wall digestion enzymes and immunolabelled with a tobacco RuBisCO polyclonal antibody^14^ to demonstrate the retention of proteins and tissue penetration of IgG antibodies for 3D whole tissue immunolocalisation. Immunolabelling of RuBisCO was observed in intact chloroplasts of both the palisade and spongy mesophyll cells (Fig. 4). Strong, nuclear localised GFP fluorescence was also observed indicating that GFP fluorescence was maintained in the correct subcellular compartment through enzyme treatment. *N. tabacum* negative controls and further examples of immunolabelling following PEA-CLARITY treatment in the model grass species, *Setaria viridis*, can be found in supplementary Figs 2 & 3 respectively.

Here PEA-CLARITY has been demonstrated as a powerful technique allowing the use of common molecular probes, including stains, endogenous fluorescent proteins and immunohistochemistry, enabling the localisation of cell components in whole unsectioned tissues. Whilst *in situ* hybridization using RNA probes has not been demonstrated here, it is theoretically possible, as demonstrated in animal tissues^4^.

As a proof of concept, mature leaves of two model species, *A. thaliana* and *N. tabacum*, were used in this study but it is anticipated that the PEA-CLARITY protocol will be applicable to a wide range of species although it may require tailoring to individual species and tissue types. Preliminary trials were conducted applying this protocol to an array of species and tissues and it was noted that some tissues, usually older or those with high sugar contents, developed a brown coloration during the clearing process (supplementary 4). Other drawbacks include: incomplete clearing of some tissues, fragility of tissue after enzymatic treatment, and fading of GFP fluorescence after extended passive clearing (~6 months or greater). We found that impure enzyme preparations may contain nucleases and proteases that degrade certain cell contents, and note that infusion of antibodies through cell walls remains difficult after incomplete enzyme digestion. Application of intermittent vacuum during enzyme and antibody incubations greatly improved penetration when compared to non-vacuum treated samples. Clearing was improved in many tissues by harvesting at the end of the dark photoperiod which reduced levels of starch.

Barriers to further clearing and penetration of larger fluorophores include tissues containing waxes and phenolics, for example, thick epidermal surfaces and lignified tissues. While no commercially available ligninases and cutinases are currently available, enzymes such as these may be developed in future and chemical treatments are being explored to potentially improve clearing of waxy and phenolic tissues. In addition, optimisation of tissue specific cell wall degrading enzyme cocktails may further enhance antibody penetration and optical clarity. The enzyme degradation step presented here in PEA-CLARITY may also be of benefit to mammalian systems, aiding in clearing and fluorophore penetration of difficult animal tissues. Also in animal tissues, light sheet microscopy has been used to reduce image acquisition times, decrease tissue bleaching, and improve z-plane resolution when generating 3D reconstructions^15^.

The adaptation of CLARITY clearing and imaging protocols to plant tissues paves the way for 3D molecular interrogation of intact plant samples. Implementation of PEA-CLARITY will allow a more complete understanding of the relationship between structure and function within plant organs and of spatially regulated molecular processes.

## Experimental Procedures

### Plant Growth Conditions

*Nicotiana tabacum* plants were grown in a glasshouse in Newcastle, Australia with supplemented sunlight for 16 hrs light and 8 hrs dark. *Arabidopsis thaliana* GFP and CFP lines C16251 and C16259^12^ were grown in a growth cabinet with 16 hr/8 hr, 23/18 °C day night cycles and photon flux of ~150 μmol. m^−2^. s^−1^ during light hours.

### Tissue Fixation and Clearing

*N. tabacum* leaf discs were excised using a 7 mm leaf punch 30 min prior to commencement of the light period thus limiting starch accumulation. Discs were then immediately drop fixed into CLARITY hydrogel solution (see supplementary 6) and kept on ice. Whole leaves of *A. thaliana* were also harvested 30 mins prior to commencement of the light period and drop fixed as above. All tissues were put under vacuum for 1 hr at −100 kPa whilst on ice to facilitate infiltration of the hydrogel. Samples were kept at 4 °C overnight in hydrogel solution. Each disc/leaf was transferred to a separate tube, filled with hydrogel, taking care to remove air bubbles before being sealed and polymerised at 37 °C overnight. Excess hydrogel was removed after polymerisation and samples transferred to 50 ml 4% SDS clearing solution buffered with boric acid (see supplementary 6). Samples were passively cleared at 37 °C with gentle agitation for 4 to 6 weeks (or until clear), changing the clearing solution daily. Once cleared, samples were stained, or those containing endogenous fluorescence were mounted for imaging.

### Staining

Cleared tobacco leaf discs were stained with 0.1% aqueous propidium iodide and/or (depending on enzyme cocktail mix) 0.05% aqueous calcofluor white for 20 mins in dark before being washed with 0.001% NaN_3_ in PBS pH 7.4.

### Enzyme Degradation

Enzymatic degradation of the cell wall was achieved by firstly washing the cleared sample with 0.001% NaN_3_ in PBS pH 7.4 with at least 3 changes to remove all SDS (SDS inhibits enzymatic activity). Once all SDS was removed samples were carefully placed into the enzyme cocktail mix containing cellulase, xylanase, arabinofuranosidase, pectate lyase and α-amylase (see supplementary 6) and vacuum infiltrated 3 × 5 mins at −100 kPa. Samples were then incubated at 37 °C with very gentle agitation in darkness. Vacuum infiltration was repeated daily for 5–7 days and samples were transferred into fresh enzyme solution after 3 days. Samples were removed from the enzyme cocktail with care, before washing 3 times with 0.001% NaN_3_ in PBS pH 7.4.

### Immunofluorescence localisation of RuBisCO

Tissues were placed into solution containing anti-RuBisCO^14^ diluted 1:100 in PBST pH 7.4 and incubated for 5 days with intermittent vacuum infiltration at −100 kPa for 3 × 5 mins, 3 × daily. Samples were then washed for 24 hrs in 50 ml PBST pH 7.4 with three solution changes and very gentle agitation. After washing, tissues were transferred into anti-rabbit Cy5 secondary antibody (1:200 in PBST pH 7.4) and incubated for 5 days with intermittent vacuum infiltration as above. Samples were washed in 0.001% NaN_3_ in PBS pH 7.4 for 24 hrs and were mounted for imaging.

### Imaging

Samples were mounted in PBS pH 7.4 and imaged with a Leica SP8 CLSM using a Leica 20 × na = 0.5 water immersion objective, white light laser and Hybrid Detectors at a resolution of 1024 × 1024 pixels. For full microscope settings (see metadata supplementary). All images displayed in this article are raw images taken from the Leica SP8. Unprocessed 3D reconstructions were performed in the Leica Applications Suite—Fluorescence (LAS-AF) software.

## Additional Information

**How to cite this article**: Palmer, W. M. *et al*. PEA-CLARITY: 3D molecular imaging of whole plant organs. *Sci. Rep*. **5**, 13492; doi: 10.1038/srep13492 (2015).

## Supplementary Material

Video - Supplementary 1

Supplementary 2,3,4

Supplementary 6

CLSM Metadata - Supplementary 5

## Figures and Tables

**Figure 1:**
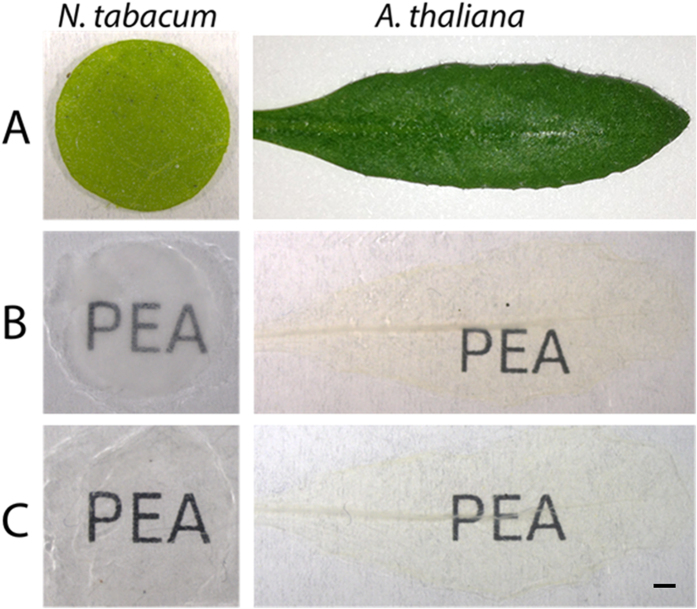
PEA-CLARITY clearing of *N. tabacum* and *A. thaliana* leaves. (**A**) fresh leaf disc from a fully expanded *N. tabacum* leaf (left) and a whole fully expanded *A. thaliana* leaf (right). (**B**) fixed, hydrogel embedded, passively cleared leaves. (**C**) cleared cell wall enzyme treated leaves for immunohistochemistry and imaging. Scale bar: 1 mm

**Figure 2:**
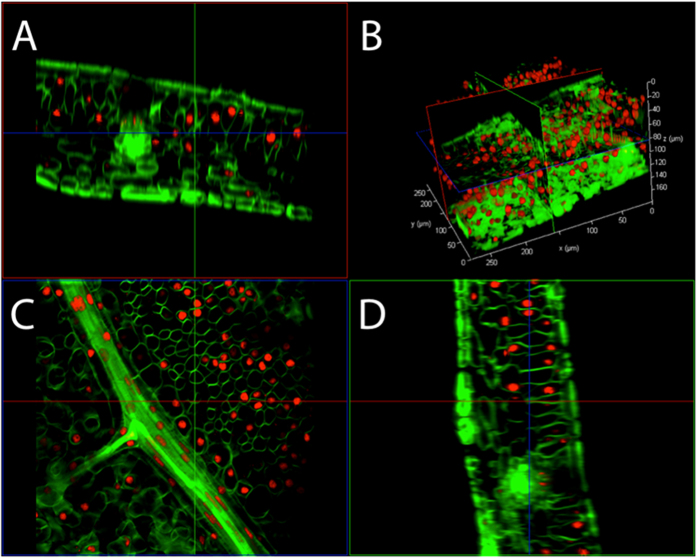
Structurally intact 3D projection of a cleared *N. tabacum* leaf showing retention of nuclear DNA. CLSM 3D projection of a passively cleared (without cell wall enzyme digestion) *N. tabacum* leaf showing nuclei stained with propidium iodide (red), and cell walls stained with calcofluor white (green). The 3D projection with epidermal layer cut away is shown in (**B**) and the x, y, z slices are shown in (**A, D)** and (**C**) respectively. The 3D video file can be viewed in supplementary 1.

**Figure 3:**
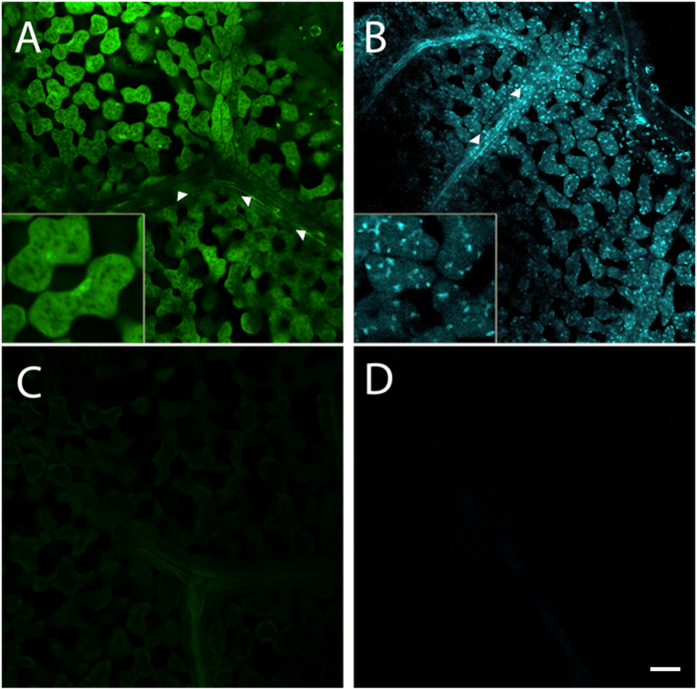
Two-dimensional optical sections of passively cleared, whole *A. thaliana* leaves showing retention of GFP and CFP fluorescence. Whole passively cleared (without cell wall enzyme treatment), whole mount *A. thaliana* leaves containing (**A**) endoplasmic reticulum localised GFP with WT negative control (**C**) and (**B**) peroxisome localised CFP with WT negative control (**D**). All were imaged through the internal phloem (arrows) of the leaf at a depth of ~80 μm. Scale bar: 50 μm

**Figure 4:**
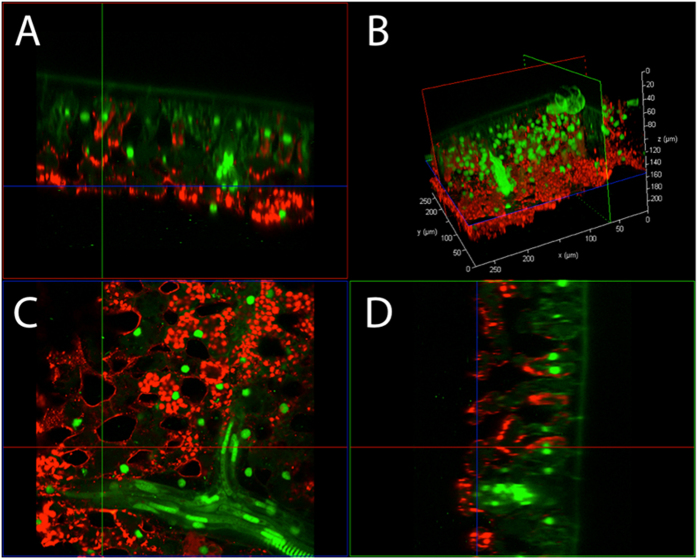
CLSM 3D projection of a PEA-CLARITY treated *N. tabacum* leaf showing immunostaining of RuBisCO and retention of GFP fluorescence. CLSM 3D projection of a passively cleared, cell wall enzyme treated (PEA-CLARITY) Sv-40 (nuclear localised GFP-green) *N. tabacum* leaf, immunostained with tobacco RuBisCO primary and Cy5 secondary antibodies (red). The 3D projection is shown in (**B**) and the x, y, z slices are shown in (**A**, **C**, **D**) respectively.
