# Supplementary material for: PEA-CLARITY: 3D molecular imaging of whole plant organs
Source: Sci Rep. 2015 Sep 2;5:13492. doi: 10.1038/srep13492 (PMC4556961; doi:10.1038/srep13492)
Supplement: Supplementary 2,3,4 [file srep13492-s2.doc]

**PEA-CLARITY: 3D molecular imaging of plant tissue**

**SUPPLEMENTARY MATERIALS**

**Supplementary 1.** Video of PI/Calcafluor stained tobacco leaf

**
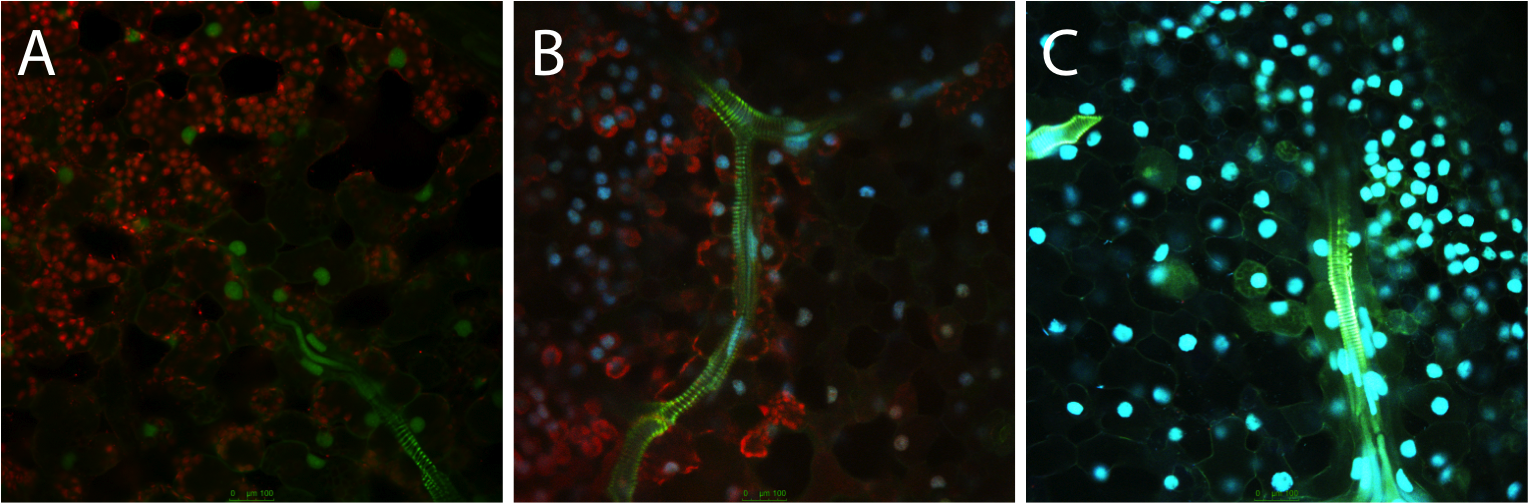
Supplementary 2. Structurally intact ‘deep’ CLSM 2D images of passively cleared PEA-CLARITY treated *N. tabacum* leaves showing immunostaining of RuBisCO and retention of GFP fluorescence with successive negative controls. A**, passively cleared, cell wall enzyme treated (PEA-CLARITY) Sv-40 (nuclear localised GFP-green) *N. tabacum* leaf, immunostained with tobacco RuBisCO primary and Cy5 secondary antibodies (red). **B**, passively cleared, cell wall treated (PEA-CLARITY) WT *N. tabacum* leaf, immunostained with tobacco RuBisCO primary and Cy5 secondary antibodies (red), and counter-stained with DAPI (cyan) showing absence of GFP expression. **C**, passively cleared, cell wall treated (PEA-CLARITY) WT *N. tabacum* leaf negative control, immunostained with Cy5 secondary antibody, but no RuBisCO primary antibody and counter-stained with DAPI (cyan). All were imaged through the internal phloem of the leaf at a depth of ~80 μm and were imaged using identical CLSM settings for Cy5 visualisation.

**
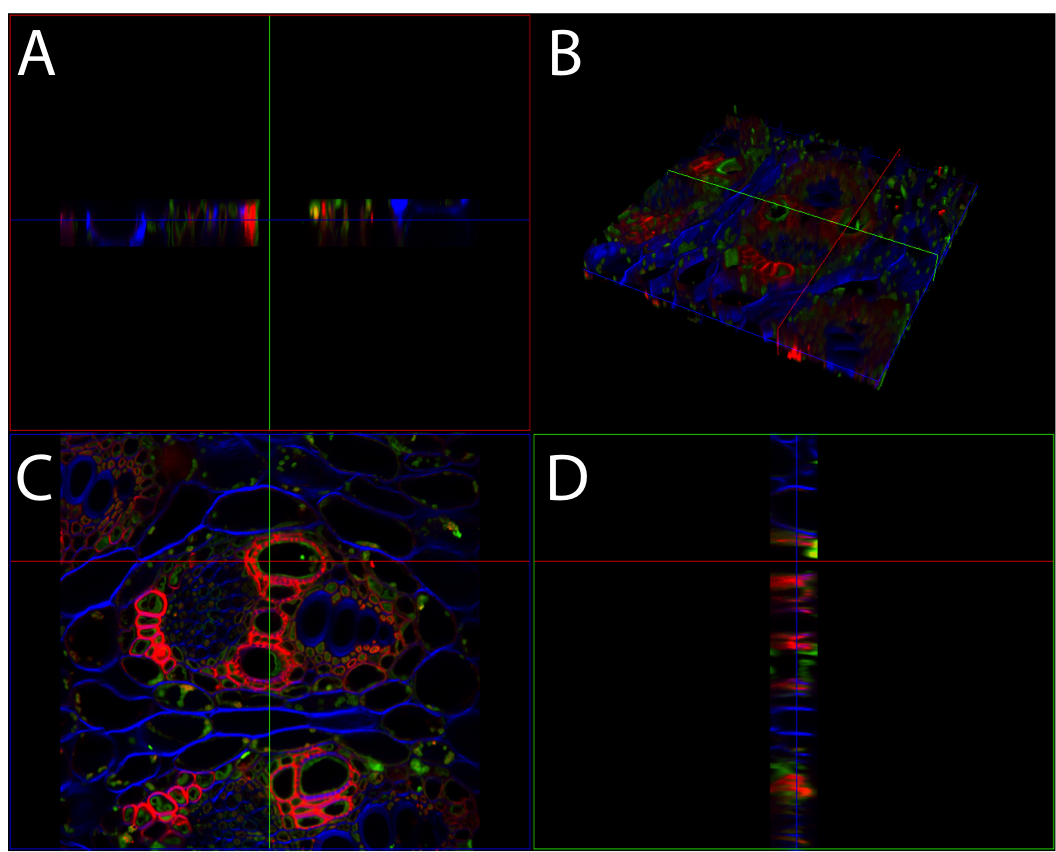
**

**Supplementary 3. CLSM 3D projection of a PEA-CLARITY treated *S. viridis* elongating stem showing dual immunostaining of H^+^-ATPase (red) and α/β-tubulin (green) with UV autofluorescence (blue).** CLSM 3D projection of a passively cleared, cell wall enzyme treated (PEA-CLARITY) *S. viridis* elongating internode, immunostained with H^+^-ATPase (raised in rabbit), α-tubulin (raised in mouse), and β-tubulin (raised in mouse) primary, and Cy5 anti-rabbit/Alexaflor488 anti-mouse secondary antibodies. H^+^-ATPase staining (red), α/β-tubulin staining (green), and UV autofluorescence are displayed. The 3D projection is shown in **B** and the x,y,z slices are shown in **D**, **A**, and **C** respectively.

**
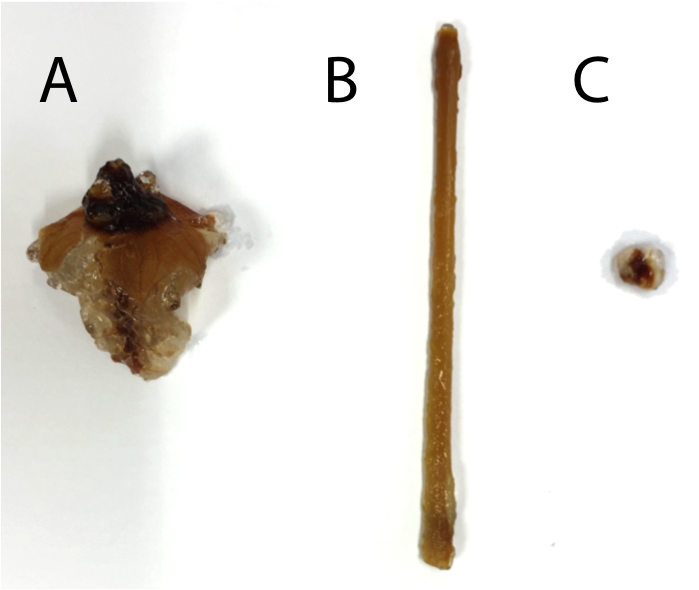
**

**Supplementary 4. Browning of plant tissues with high sugar contents during extended passive clearing. A**, *Gossypium hirsutum* (cotton) boll, **B**, fully elongated *Setaria viridis* stem internode, and **C**, *Vitis Vinifera* (grape) axillary bud, all passively cleared for an extended period (approximately 6 months). Brown coloration developed over time and impeded CLSM penetration into the tissue.

**Supplementary 5.** Metadata
