# Supplementary material for: PEA-CLARITY: 3D molecular imaging of whole plant organs
Source: Sci Rep. 2015 Sep 2;5:13492. doi: 10.1038/srep13492 (PMC4556961; doi:10.1038/srep13492)
Supplement: Supplementary 6 [file srep13492-s3.docx]

**PEA-CLARITY PROTOCOL**

William M. Palmer^1^, Antony P. Martin^1^, Jamie R. Flynn^2^, Stephanie Reed^1^, Rosemary White^3^, Robert T. Furbank^4^, Christopher P.L. Grof^1^

^1^School of Environmental and Life Sciences, University of Newcastle, Callaghan, NSW 2308, Australia.

^2^School of Biomedical Sciences and Pharmacy, University of Newcastle, Callaghan, NSW 2308, Australia.

^3^CSIRO Agriculture, Black Mountain, ACT 2601, Australia.

^4^ARC Centre of Excellence for Translational Photosynthesis, Australian National University, Acton, ACT, 2601, Australia.

*****Caution:** Please refer to MSDS before conducting protocol as paraformaldehyde (PFA), acrylamide, sodium dodecyl sulfate and sodium azide (NaN_3_) are known irritants, sensitizers, carcinogens and neurotoxins. The use of personal protective equipment (PPE) is imperative whilst undertaking this protocol.

**Plant Harvesting:**

Harvest mature, fully expanded leaves at the end of the dark period to minimise starch accumulation.

**Fixation:**

1. Excise ~20 *N.tabacum* 7 mm leaf disks of each line from fully expanded leaves before immediately drop fixing into 50 ml ice cold hydrogel solution (see reagents list).

2. Place tissue under vacuum at -100 kPa (in fume hood) for 1-2 hours on ice and in darkness if fluorophores are present to facilitate infiltration of hydrogel and removal of gas.

3. Transfer to a 4°C fridge overnight.

**Hydrogel Polymerization**:

1. Carefully remove individual leaf disks from the 50 ml conical tube and place into 1.5 ml microfuge tubes containing 1 ml fresh chilled hydrogel solution and keep on ice.

2. Place samples under vacuum for 15 min to remove excess gas from the transferring of samples.

3. Completely fill 1.5 ml tubes with hydrogel solution taking care to remove any air bubbles before sealing with parafilm.

4. Float sealed tubes in a 37°C water bath overnight to polymerize.

**Tissue Clearing:**

1. Polymerized leaf disks were removed from 1.5 ml tubes and excess hydrogel separated carefully from the sample with lint free paper.

2. Samples were washed in 50 ml of SDS clearing solution (see reagents list) 3 times daily for 2 days to remove excess unbound PFA and acrylamide at room temperature. Take care to dispose of this solution correctly.

3. 50 ml of SDS clearing solution was replaced daily for a period of 4-6 weeks (or until clear) at 37°C with very gentle agitation.

**Enzyme Treatment:**

1. Samples were extensively washed in 50 ml NaN_3_ PBS (see reagents list) with 3 changes daily for 3 days at room temperature.

*** SDS is an inhibitor of enzymatic activity and improper washing will result in incomplete enzymatic degradation of the cell wall.

2a. Samples were transferred to 1.5 ml Protein LoBind tubes containing 1 ml of Enzyme Mix (EM, see reagents list) and kept at 37°C for 5-7 days with very gentle agitation.

2b. During enzyme treatment a vacuum of -100 kPa was applied in 3 x 5 min bursts, 3 x daily to help facilitate enzyme infiltration.

2c. Fresh EM was applied on the third day.

3. Samples were very carefully removed before being washed 3 x in 50 ml PBST (see reagents list) for 24 h.

**Immunolocalization:**

1. Dilute primary/secondary antibodies to desired concentration in PBST.

2a. Transfer sample into 1.5 ml Protein LoBind tube containing 1 ml desired primary antibody/s concentration at 37°C for 5 days with very gentle agitation.

2b. During antibody treatment a vacuum of -100 kPa was applied in 3 x 5 min bursts, 3 x daily to help facilitate primary antibody infiltration.

3. Wash 3 x 50 ml PBST for 24 h

4. Follow 2a and 2b above for secondary antibody/s

5. Wash 3 x 50 ml NaN_3_ PBS for 24 h.

**Imaging Preparation:**

*** During this protocol samples were mounted in PBS however, as described in text, other mounting mediums such as Focus Clear and RIMS can be used to optically match the hydrogel. If using other mounting mediums then samples will need to be incubated prior to mounting.

1. Using a small piece of BluTack create a well by rolling the putty into a long cylindrical shape and apply to a glass microscope slide so the sides of the well are just higher than the sample thickness (~2 mm).

2. Seal the outer rim of the BluTack well and the microscope slide with a thin layer of Vaseline.

3. Half fill well with PBS (or mounting medium).

4. Place sample into well and cover with PBS (or mounting medium).

5. Place a glass microscope coverslip over the well ensuring there are no air bubbles.

6. Samples are now ready for imaging.

**PEA-CLARITY REAGENTS**

| **REAGENT** | **COMPANY** | **CATALOGUE NUMBER** |
| --- | --- | --- |
| Phosphate Buffered Saline | Gibco | 21600-010 |
| 40% Acrylamide | Biorad | 161-0140 |
| 2% Bis Acrylamide | Biorad | 161-0142 |
| 16% Paraformaldehyde | Electron Microscopy Sciences | 15710 |
| VA-044 Initiator | Wako | 017-19362 |
| Sodium Dodecyl Sulfate | Sigma | L-3771 |
| Boric Acid | Sigma | B-6768 |
| Sodium Hydroxide | Sigma | S-8045 |
| Triton-X | Sigma | T-9284 |
| Sodium Azide | Sigma | S-2002 |
| Propidium Iodide | Sigma | P-4864 |
| Calcofluor White | Sigma | F-3543 |
| Rubisco Antibody (rabbit) | Gift | Spencer Whitney |
| Cy5 Secondary AB (anti-rab) | Abcam | Ab6564 |
| α-Amylase | Megazyme | E-ANAAM |
| α-L-Arabinofuranosidase | Megazyme | E-ABFCJ |
| β-Mannanase | Megazyme | E-BMACJ |
| Cellulase | Megazyme | E-CELBA |
| Pectate Lyase | Megazyme | E-PLYCJ |
| Xyloglucanase | Megazyme | E-XEGP |
| Calcium Chloride | Sigma | C-5670 |

**HYDROGEL SOLUTION (200 ml)**

| **Reagent** | **Amount** | **Concentration** |
| --- | --- | --- |
| 40% Acrylamide | 20 ml | 4% |
| 2% Bis Acrylamide | 5 ml | 0.05% |
| VA-044 Initiator | 500 mg | 0.25% |
| 10 x PBS pH 7.4 | 20 ml | 1 x |
| 16% Paraformaldehyde | 50 ml | 4% |
| MQ-H_2_O | 105 ml | - |

*****Store at 4°C**

**SDS CLEARING SOLUTION (1 L)**

| **Reagent** | **Amount** | **Concentration** |
| --- | --- | --- |
| Boric Acid | 12.366 g | 200 mM |
| SDS | 40 g | 4% |
| MQ-H_2_0 | Fill to 1 L | - |
| NaOH | To pH 8.5 | - |

**ENZYME TREATMENT SOLUTION (10 ml)**

| **Reagent** | **Amount** | **Concentration** |
| --- | --- | --- |
| α-Amylase | 10 µl | 10 U |
| α-L-Arabinofuranosidase | 10 µl | 5 U |
| β-Mannanase | 10 µl | 50 U |
| Cellulase | 10 µl | 14 U |
| Pectate Lyase | 10 µl | 6.61 U |
| Xyloglucanase | 10 µl | 10 U |
| Calcium Chloride | 1.1 mg | 1 mM |
| 1 x PBS pH 7.4 (0.005% NaN_3_) | 9.94 ml | - |
